# Supplementary material for: Integrative taxonomy revisits the ontogeny and trophic niches of Rimicaris vent shrimps
Source: R Soc Open Sci. 2020 Jul 8;7(7):200837. doi: 10.1098/rsos.200837 (PMC7428246; doi:10.1098/rsos.200837)
Supplement: Supplementary Materials with figures and tables [file rsos200837supp1.pdf]

Supplementary Materials

Morphological analysis: Determination of Onset of Sexual Differentiation (OSD) in *R. chacei*

Since subadult shrimps are morphologically similar to small females, i.e., they lack male morphological characteristics of appendix masculina on the second pleopod and modified endopod on first pleopod, dissection procedures to assess the presence of gonadal tissue were performed to separate sexually mature females from subadults. This makes it possible to identify an average shrimp size for the onset of sexual differentiation (OSD). The OSD was used as a proxy to differentiate subadults from small adults. For *R. exoculata*, this size is 9.9 mm, as estimated by a previous study conducted on individuals collected from the same vent sites in the same period of the year (Hernández-Ávila 2016). To obtain a similar estimation for *R. chacei*, a sample of 10 to 20 shrimps per size class, ranging from 4 mm to 12 mm in CL, were collected during the BICOSE 2 cruise. A total of 130 individuals were dissected to verify macroscopic evidence of gonadal tissue. OSD was calculated according to (Hernández-Ávila 2016; Wenner, Fusaro, and Oaten 1974) (see fig. S2).

Genetic identification: PCR amplification protocol

PCR amplifications were conducted with 1 µl of template DNA in a 50 µL solution of 1X reaction buffer, 2 mM $MgCl_2$, 0.4 µM dNTP, 0.6 units of Taq polymerase and 0.6 mM of each primer. PCR cycles were performed as follows: initial denaturation (5 min at 94°C), 35 cycles including denaturation (1 min at 94°C), annealing (1 min at 50 °C) and elongation (2 min at 72 °C), followed by a final elongation of 7 min at 72°C. All PCR amplifications were conducted on a GeneAmp PCR system 9700 (Applied Biosystems). PCR products that produced visible bands under UV light after electrophoresis on 1% agarose gel were sent to the MacroGen Europe Laboratory in Amsterdam (The Netherlands) to obtain sequences, using the same set of primers as used for the PCR. Overlapping sequence (forward and reverse) fragments were merged into a consensus sequence using Geneious Pro 8.1.9 (Biomatters Ltd). The minimal length of all COI sequences coverage was 612 bp.

Statistical analysis and data processing: isotopic niche analysis

The SIBER approach involves the use of ellipses (Jackson et al. 2011) to define isotopic niches, i.e., the space occupied by an animal population in a bivariate isotopic space. Since variation in the isotopic composition of animals (i.e., position of points in the isotopic space) is dictated by both the prey items consumed (Jackson et al. 2011; McCutchan et al. 2003) and habitat use (Flaherty and Ben-David 2010), this isotopic niche can be used as a proxy of the realized ecological niche. Overlap between ellipses associated with different populations suggests that these groups partly exploit the same food and/or habitat resources. The bigger the overlap, the more resources are shared by the two populations. Here, we used standard ellipses (bivariate equivalent of standard deviations). These standard ellipses contain only the "typical" members of a population (but may not encompass outlier individuals in the isotopic space). For this reason, they are termed "core isotopic niches", as they can be used as proxies of the trophic and habitat resources most commonly used by populations (Layman and Allgeier 2012).

Supplementary figures and tables caption

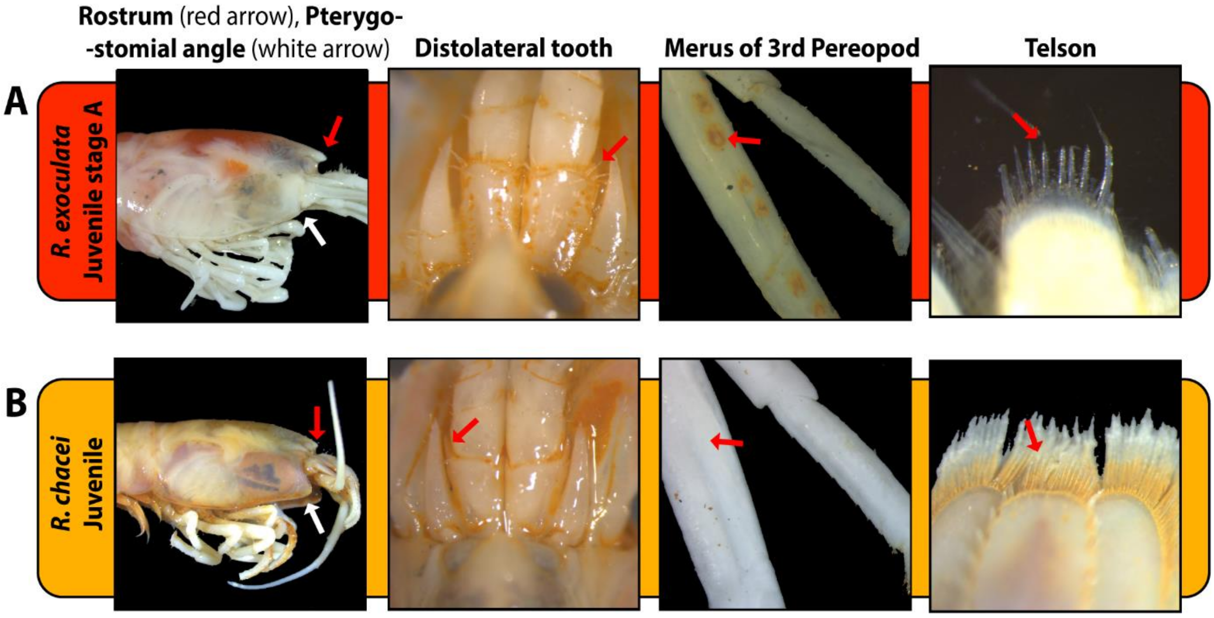

Figure S1. Summary of the morphological characters used to assign morphotypes of small alvinocaridid juveniles from (Komai and Segonzac 2008). Each character is indicated by an arrow. **A.***R. exoculata* juvenile stage A: dorsally triangular produced rostrum, pterygostomial angle of the carapace straight or very slightly produced, absent or small and partially obscured distolateral tooth on the first segment of the antennular peduncle, spiniform setae on the posterior margin of the telson, and meri of second and third pereopods armed with spines. **B.** *R. chacei* juvenile: rounded and only slightly produced rostrum, pterygostomial angle of the carapace produced anteriorly, conspicuous distolateral tooth on the first segment of the antennular peduncle, plumose setae on the posterior margin of the telson and meri of second and third pereopods unarmed.

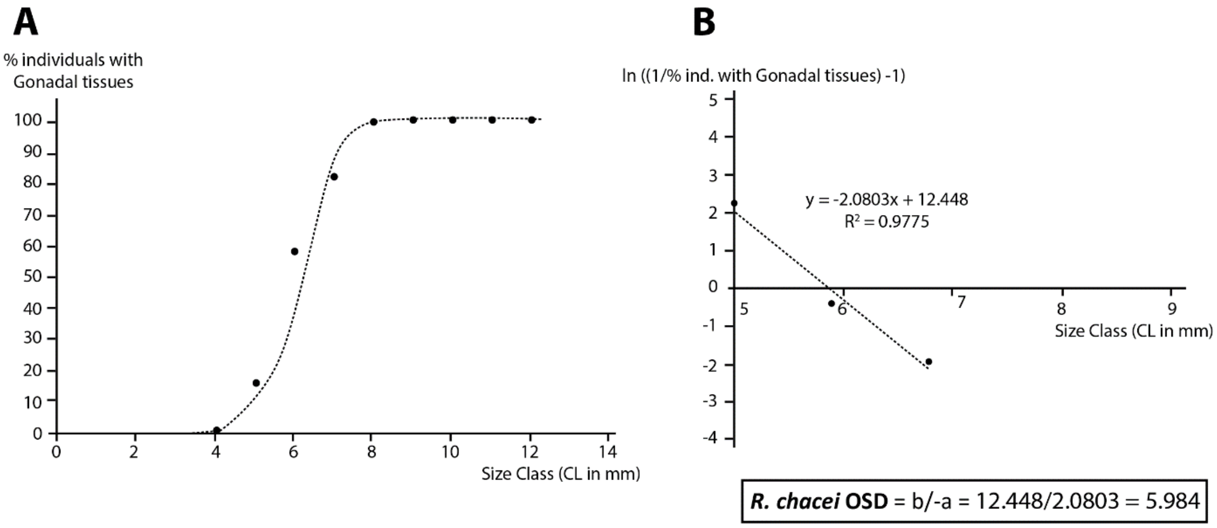

Figure S2. Estimation of *R. chacei* sexual maturity to determine its Onset of Sexual Differentiation (OSD). **A.** Proportion of individuals with developed gonadal tissues per size class. **B.** Log-transformed proportion of individuals with developed gonadal tissues per size class.

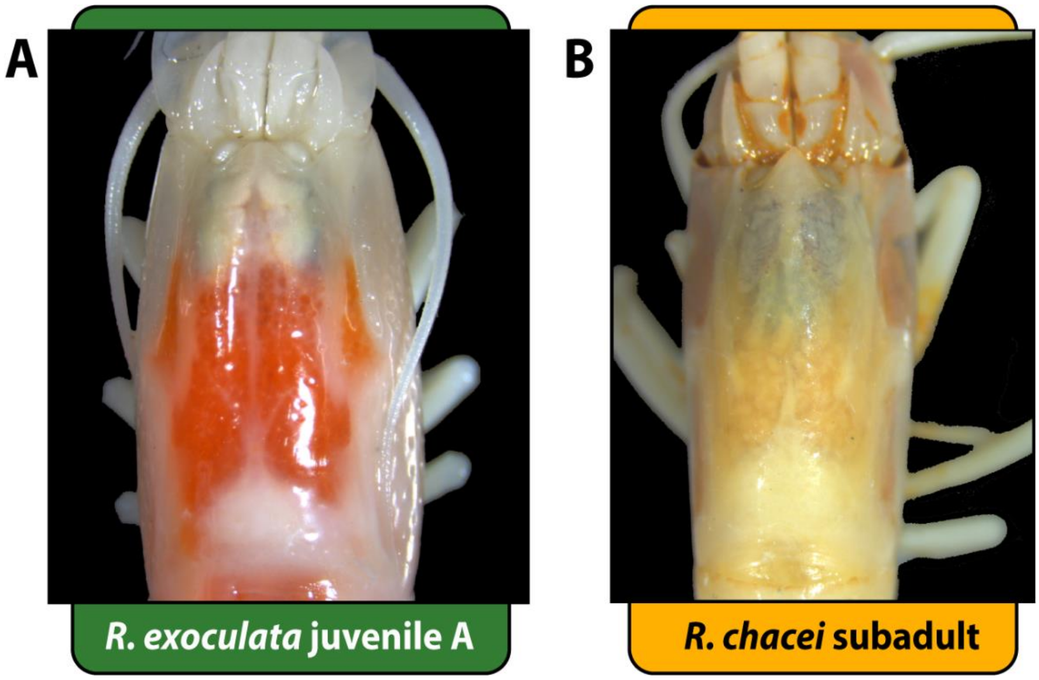

Figure S3. Morphological characters used to distinguish **A.** *Rimicaris exoculata* stage A juveniles from **B.** *Rimicaris chacei* subadults. General shape of the carapace: **A.** not inflated but tapered on the anterior part; **B.** not inflated and straight on the anterior part. Amount of the red/orange lipid reserves: **A.** important **B.** limited.

**A.**	$\delta^{13}C$	$\delta^{15}N$	$\delta^{34}S$
**Life stages comparisons (Kruskal-Wallis test):**			
R. exoculata life stages (TAG)	**9,51E-11**	**1,31E-09**	**4,30E-11**
R. exoculata life stages (Snake Pit)	**3,31E-10**	**1,58E-09**	**1,93E-10**
R. chacei life stages (TAG)	**1,72E-07**	**1,26E-08**	**1,28E-08**
R. chacei life stages (Snake Pit)	**5,94E-04**	**1,41E-02**	**4,27E-07**
**Inter species (*R. exoculata* vs *R. chacei*) comparisons (Wilcoxon test):**			
Adults (TAG)	**2,20E-16**	**1,17E-03**	**2,20E-16**
Adults (Snake Pit)	**8,73E-09**	**5,84E-03**	1,57E-01
Subadults (TAG)	**1,51E-03**	3,93E-01	8,53E-01
Subadults (Snake Pit)	**4,33E-02**	7,39E-01	1,66E-01
Stage A juveniles (TAG)	**7,88E-04**	9,18E-01	8,09E-01
Stage A juveniles (Snake Pit)	**4,33E-05**	7,39E-01	3,15E-01

**B.**	$\delta^{13}C$			$\delta^{15}N$			$\delta^{34}S$		
***R. exoculata* life stages (TAG) detailed comparisons (pairwise wilcoxon post hoc tests):**									
	Adults	Subadults	Stage B juveniles	Adults	Subadults	Stage B juveniles	Adults	Subadults	Stage B juveniles
Subadults	**2,3947E-03**			**2,8000E-08**			**4,7000E-09**		
Stage B juveniles	**1,7369E-06**	1,8124E-01		**1,4000E-07**	9,1000E-01		**4,7000E-09**	**1,1000E-03**	
Stage A juveniles	**1,3535E-08**	5,1588E-02	5,1843E-01	**3,8000E-09**	1,7000E-01	3,0000E-01	**3,8000E-09**	**8,5000E-06**	**4,8000E-05**
***R. exoculata* life stages (Snake Pit) detailed comparisons (pairwise wilcoxon post hoc tests):**									
	Adults	Subadults	Stage B juveniles	Adults	Subadults	Stage B juveniles	Adults	Subadults	Stage B juveniles
Subadults	**1,6000E-08**			**1,6000E-08**			**1,6000E-08**		
Stage B juveniles	**1,6000E-08**	**5,8000E-04**		**1,6000E-08**	9,0300E-02		**1,6000E-08**	**1,3000E-04**	
Stage B juveniles	**1,6000E-08**	**1,6000E-05**	**5,2000E-03**	**1,6000E-08**	**3,1000E-03**	1,2300E-01	**1,6000E-08**	**2,6000E-05**	**2,6000E-05**

**C.**	$\delta^{13}C$		$\delta^{15}N$		$\delta^{34}S$	
***R. chacei* life stages (TAG) detailed comparisons (pairwise wilcoxon post hoc tests):**						
	Adults	Subadults	Adults	Subadults	Adults	Subadults
Subadults	**7,9000E-05**		**4,7000E-08**		**7,7000E-07**	
Stage A juveniles	**2,2000E-08**	**2,9000E-03**	**3,1000E-09**	**1,5000E-02**	**3,1000E-09**	**1,1000E-05**
***R. chacei* life stages (Snake Pit) detailled comparison (pairwise wilcoxon post hoc test):**						
	Adults	Subadults	Adults	Subadults	Adults	Subadults
Subadults	3,3180E-01		1,0800E-01		**1,5000E-05**	
Stage A juveniles	**7,0000E-04**	**3,9000E-04**	**2,2000E-02**	1,9000E-01	**4,6000E-07**	**1,3000E-04**

Table S1. p-values of the statistical tests used for isotopic ratio comparisons ($\delta^{13}C$, $\delta^{15}N$ $\delta^{34}S$). **A.** Kruskal-Wallis p-values for overall life stage comparisons in each species for a given vent field, and Wilcoxon p-values for interspecies comparisons at equivalent life stage at each vent field. **B.** Pairwise Wilcoxon p-values (Kruskal-Wallis post-hoc tests) for life stage comparisons in *R. exoculata* at TAG or at Snake Pit. **C.** Pairwise Wilcoxon p-values (Kruskal-Wallis post-hoc tests) for life stage comparisons in *R. chacei* at TAG or at Snake Pit.

**A.**	**δ^{13}C vs δ^{15}N ellipses**												
	***R. exoculata* adults TAG**	***R. exoculata* adults Snake Pit**	***R. exoculata* subadults TAG**	***R. exoculata* subadults Snake Pit**	***R. exoculata* stage B juveniles TAG**	***R. exoculata* stage B juveniles Snake Pit**	***R. exoculata* stage A juveniles TAG**	***R. exoculata* stage A juveniles Snake Pit**	***R. chacei* adults TAG**	***R. chacei* adults Snake Pit**	***R. chacei* subadults TAG**	***R. chacei* subadults Snake Pit**	***R. chacei* stage A juveniles TAG**
***R. exoculata* adults Snake Pit**	0.01 ‰^{2}												
***R. exoculata* subadults TAG**	no	no											
***R. exoculata* subadults Snake Pit**	no	no	0.23 ‰^{2}										
***R. exoculata* stage B juveniles TAG**	no	no	0.04 ‰^{2}	no									
***R. exoculata* stage B juveniles Snake Pit**	no	no	0.32 ‰^{2}	no	no								
***R. exoculata* stage A juveniles TAG**	no	no	no	no	0.06 ‰^{2}	no							
***R. exoculata* stage A juveniles Snake Pit**	no	no	no	no	0.11 ‰^{2}	0.05 ‰^{2}	0.22 ‰^{2}						
***R. chacei* adults TAG**	no	no	no	no	no	no	no	no					
***R. chacei* adults Snake Pit**	no	no	1.6 ‰^{2}	0.32 ‰^{2}	0.61 ‰^{2}	0.45 ‰^{2}	0.18 ‰^{2}	0.23 ‰^{2}	0.37 ‰^{2}				
***R. chacei* subadults TAG**	no	no	0.24 ‰^{2}	no	0.57 ‰^{2}	0.07 ‰^{2}	0.52 ‰^{2}	0.16 ‰^{2}	no	0.88 ‰^{2}			
***R. chacei* subadults Snake Pit**	no	no	1.4 ‰^{2}	0.27 ‰^{2}	0.21 ‰^{2}	0.48 ‰^{2}	0.19 ‰^{2}	0.41 ‰^{2}	no	1.97 ‰^{2}	0.45 ‰^{2}		
***R. chacei* stage A juveniles TAG**	no	no	no	no	no	no	no	no	no	no	no	no	
***R. chacei* stage A juveniles Snake Pit**	no	no	no	no	no	no	0.02 ‰^{2}	no	no	no	0.06 ‰^{2}	no	0.36 ‰^{2}

**B.**	**δ^{13}C vs δ^{34}S ellipses**												
	***R. exoculata* adults TAG**	***R. exoculata* adults Snake Pit**	***R. exoculata* subadults TAG**	***R. exoculata* subadults Snake Pit**	***R. exoculata* stage B juveniles TAG**	***R. exoculata* stage B juveniles Snake Pit**	***R. exoculata* stage A juveniles TAG**	***R. exoculata* stage A juveniles Snake Pit**	***R. chacei* adults TAG**	***R. chacei* adults Snake Pit**	***R. chacei* subadults TAG**	***R. chacei* subadults Snake Pit**	***R. chacei* stage A juveniles TAG**
***R. exoculata* adults Snake Pit**	0.02 ‰^{2}												
***R. exoculata* subadults TAG**	no	no											
***R. exoculata* subadults Snake Pit**	no	no	0.85 ‰^{2}										
***R. exoculata* stage B juveniles TAG**	no	no	0.02 ‰^{2}	no									
***R. exoculata* stage B juveniles Snake Pit**	no	no	0.93 ‰^{2}	no	0.07 ‰^{2}								
***R. exoculata* stage A juveniles TAG**	no	no	no	no	no	no							
***R. exoculata* stage A juveniles Snake Pit**	no	no	no	no	no	no	0.24 ‰^{2}						
***R. chacei* adults TAG**	no	no	no	no	no	no	no	no					
***R. chacei* adults Snake Pit**	no	no	no	no	no	no	no	no	0.01 ‰^{2}				
***R. chacei* subadults TAG**	no	no	no	no	no	no	no	no	no	no			
***R. chacei* subadults Snake Pit**	no	no	1.05 ‰^{2}	0.36 ‰^{2}	no	0.45 ‰^{2}	no	no	1.65 ‰^{2}	0.19 ‰^{2}	1.22 ‰^{2}		
***R. chacei* stage A juveniles TAG**	no	no	no	no	no	no	0.02 ‰^{2}	no	no	no	no	no	
***R. chacei* stage A juveniles Snake Pit**	no	no	no	no	0.05 ‰^{2}	no	0.27 ‰^{2}	no	no	no	no	no	0.93 ‰^{2}

Table S2. Values of the overlap areas between isotopic ellipses of each life stage of *R. exoculata* and *R. chacei* at each of the two vent fields. **A.** for δ^{13}C vs δ^{15}N ellipses and **B.** for δ^{13}C vs δ^{34}S ellipses. no: absence of overlap.

References

Flaherty, Elizabeth A., and Merav Ben-David. 2010. "Overlap and Partitioning of the Ecological and Isotopic Niches." *Oikos* 119 (9): 1409–16. https://doi.org/10.1111/j.1600-0706.2010.18259.x.

Hernández-Ávila, I. 2016. "Larval Dispersal and Life Cycle in Deep-Water Hydrothermal Vents : The Case of *Rimicaris exoculata* and related species." PhD Thesis. Université de Bretagne Occidentale.

Jackson, Andrew L., Richard Inger, Andrew C. Parnell, and Stuart Bearhop. 2011. "Comparing Isotopic Niche Widths among and within Communities: SIBER - Stable Isotope Bayesian Ellipses in R." *Journal of Animal Ecology* 80 (3): 595–602. https://doi.org/10.1111/j.1365-2656.2011.01806.x.

Komai, Tomoyuki, and Michel Segonzac. 2008. "Taxonomic Review of the Hydrothermal Vent Shrimp Genera *Rimicaris* Williams & Rona and *Chorocaris* Martin & Hessler (Crustacea: Decapoda: Caridea: Alvinocarididae)." *Journal of Shellfish Research* 27 (1): 21–41. https://doi.org/10.2983/0730-8000(2008)27[21:TROTHV]2.0.CO;2.

Layman, Craig A, and Jacob E Allgeier. 2012. "Characterizing Trophic Ecology of Generalist Consumers : A Case Study of the Invasive Lionfish in The Bahamas." *Marine Ecology Progress Series* 448: 131–41. https://doi.org/10.3354/meps09511.

McCutchan, James H., William M. Lewis, Carol Kendall, and Claire C. McGrath. 2003. "Variation in Trophic Shift for Stable Isotope Ratios of Carbon, Nitrogen, and Sulfur." *Oikos* 102 (2): 378–90. https://doi.org/10.1034/j.1600-0706.2003.12098.x.

Wenner, M. Adrian, Craig Fusaro, and Allan Oaten. 1974. "Size at Onset of Sexual Maturity and Growth Rate in Crustacean Populations." *Canadian Journal of Zoology* 52 (9): 1095–1106. https://doi.org/10.1890/06-0562.1.PMID.
